# The histone demethylase JMJD2A promotes glioma cell growth via targeting Akt-mTOR signaling

**DOI:** 10.1186/s12935-020-01177-z

**Published:** 2020-03-30

**Authors:** Min Li, Jingmin Cheng, Yuan Ma, Heng Guo, Haifeng Shu, Haidong Huang, Yongqin Kuang, Tao Yang

**Affiliations:** Department of Neurosurgery, General Hospital of Western Theater Command, Chengdu, No. 270, Rongdu Avenue, Jinniu District, Chengdu, China

**Keywords:** JMJD2A, Glioma, mTOR, Rapamycin, PDK1

## Abstract

**Background:**

A number of JmjC domain-containing histone demethylases have been identified and biochemically characterized in mammalian models and humans. JMJD2A is a transcriptional co-factor and enzyme that catalyzes the demethylation of histone H3 lysine 9 and 36 (H3K9 and H3K36). Here in this study, we reported the role of JMJD2A in human glioma.

**Methods:**

Quantitative real-time PCR and western blot were performed to analyzed JMJD2A expression in glioma. Log-rank was performed to plot the survival curve. JMJD2A was knocked or overexpressed with lentivirus. Cell proliferation and colony formation were performed to assess the effects of JMJD2A on glioma cell growth. Xenograft experiment was performed the evaluate the growth rate of glioma cells in vivo. The signaling pathway was analyzed with western blot and mTOR was inhibited with rapamycin.

**Results:**

Quantitative real-time PCR and western blot experiments revealed higher expression of JMJD2A and lower levels of H3K9me3/H3K36me3 in glioma tissues than that in normal brain tissues. We showed that knockdown of *JMJD2A* expression attenuated the growth and colony formation in three lines of glioma cells (U251, T98G, and U87MG), whereas *JMJD2A* overexpression resulted in opposing effects. Furthermore, we performed in vivo xenograft experiments and our data demonstrated that *JMJD2A* knockdown reduced the growth of glioma T98G cells in vivo. Further mechanism study implicated that *JMJD2A* activated the Akt-mTOR pathway and promoted protein synthesis in glioma cells via promoting phosphoinositide-dependent kinase-1 (*PDK1)* expression. The activation of the Akt-mTOR pathway was also validated in human glioma tissues. Finally, we showed that inhibition of mTOR with rapamycin blocked the effects of *JMJD2A* on protein synthesis, cell proliferation and colony formation of glioma cells.

**Conclusions:**

These findings demonstrated that JMJD2A regulated glioma growth and implicated that JMJD2A might be a promising target for intervention.

## Background

High-grade gliomas, which include glioblastomas and diffuse intrinsic pontine gliomas, represent a highly malignant type of brain tumor [1]. Similar to other cancers, comprehensive sequencing studies have revealed a variety of genetic abnormalities in chromatin remodeling factors in gliomas [2]. The mutation of histone H3 was reported to account for some gliomas [3–5]. However, the roles of histone methylases or demethylases in gliomas are not fully understood.

The JMJD (JmjC domain-containing) proteins, is composed of 30 members in humans based on the presence of the roughly 150 amino acid–long JmjC domain [6]. Among this family, JMJD2A, JMJD2B, and JMJD2C are overexpressed in breast, colorectal, lung, prostate, and other tumors and are required for efficient cancer cell growth [7]. The most studied member of the JMJD2 family may be JMJD2A. A major of studies focus on JMJD2A has been in transcription regulation, where it may either stimulate or repress gene transcription. The latter function of JMJD2A involves association with the nuclear receptor co-repressor complex or histone deacetylases or binding directly to a transcription factor such as the p53 tumor suppressor [8–10]. Diverse physiological or pathological functions of JMJD2A have been identified in human subjects and animal models. Recently, JMJD2A has been reported to participate in several types of cancer, including duct carcinoma [11], breast cancer [12, 13], lung cancer [14, 15], colon cancer [10], bladder cancer [16], ovarian cancer, renal adenocarcinoma, and head and neck squamous cell carcinoma [17]. The aim of this study is to determine the role of JMJD2A in human glioma and the underlying mechanism.

Here in the present study, we provided evidence that JMJD2A was up-regulated in human glioma tissues and JMJD2A promoted glioma cell growth by promoting the activation of the Akt-mTOR pathway. Our data implicate that JMJD2A could be a potential therapeutic target for glioma.

## Materials and methods

### Human glioma

Nineteen cases of patients with glioma with full case history between June 2005 and October 2009 were selected and included in the present study. Fresh glioma tissues were obtained, and the fresh tissues were stored at − 80 °C untile use. All human tissue samples of normal brain and glioma were obtained from the General Hospital of Western Theater Command *(Chengdu, China)*. All samples were classified according to the fourth edition of the histological grades of tumors of the nervous system published by the WHO in 2007 [1]. A written form of informed consent was obtained from all patients and donors. The study was approved by the *Clinical Research Ethics Committee of* Southern Medical University.

We also analyzed the expression of JMJD2A and its correlation with overall survival in glioma using the TCGA database with the web tool GEPIA2 (http://gepia2.cancer-pku.cn).

### Cell lines and cell culture

The NHA cell line was purchased from the Lonza group and cultured with Clonetics medium and reagents. Human glioma cell lines T98G, U87MG, A172, U251, and CCF-STTG1 were purchased from the ATCC and cultured according to the guidelines recommended by the ATCC. All cells were maintained at 37 °C with 5% CO_2_. For drug treatment of cells, the mTOR inhibitor rapamycin (MedChemExpress, HY-10219) and PDK1 inhibitor OSU-03012 (Selleck, S1106) were used.

### Western blot

Fresh tissues and cells were lysed with cell lysis buffer (Beyotime Biotechnology) and western blot was performed as described previously [18]. Briefly, 40 μg total proteins were applied to separation with SDS–PAGE gel. After the electrophoresis, the proteins were transferred to PVDF membranes (Millipore), followed by blocking in the TBST buffer containing 5% fat-free milk. The membranes were then incubated with indicated antibodies overnight at 4 °C, and then washed and incubated with HRP-conjugated secondary antibodies (Zhongsanjinqiao) for 2 h at room temperature and finally visualized using Chemiluminescent ECL reagent (Vigorous Biotechnology). The following antibodies were used in this work: Anti-GAPDH (Cell Signaling Technology), anti-JMJD2A (Cell Signaling Technology), anti-Histone H3 (Santa Cruz Biotechnology), anti-H3K9me3 (Abcam), anti-H3K36me3 (Abcam), anti-mTOR (Cell Signaling Technology), anti-p-mTOR (Cell Signaling Technology), anti-Akt (Cell Signaling Technology), anti-p-Akt Thr308 (Cell Signaling Technology), anti-S6K1 (Cell Signaling Technology), anti-p-S6K1 (Cell Signaling Technology).

### Quantitative real-time PCR (qRT-PCR)

Total RNA was extracted from cells or tissues with TRIzol (ThermoFisher) and cDNA was synthesized from one μg of total RNA with one-step RT-PCR Kit (TaKaRa). qRT-PCR was performed with the SYBR Green detection method on an ABI-7500 RT-PCR system (Applied Biosystems) with the SYBR Green qRT-PCR kit (TaKaRa). GAPDH was used as a control housekeeping gene. The primers used were listed as: JMJD2A forward: 5′-CCAGAACCAACCAGGAGC-3′ JMJD2A reverse: 5′-TTCACT GCGCGAGACCAT-3′ GAPDH forward: 5′-TATGATGATATCAAGAGGGTAG-3′ GAPDH reverse: 5′-ACTTTGACAATAACTGTCC-3′.

### Lentivirus packaging

Sh-*JMJD2A* and control shRNA (sh-Ctrl) lentivirus particles were purchased from GenePharma. The sh-*JMJD2A* sequence is: 5′-GCCACGAGCATCCTATGATGA-3′. Lentivirus expressing human *JMJD2A* was generated by sub-cloning human JMJD2A cDNA (NM_014663.2) to the pSLIK lentivirus expression system. For lentiviral packaging, HEK293T cells were co-transfected with the lentiviral particles. For transduction, cells were incubated with virus-containing supernatant in the presence of 5 µg/ml polybrene. After 48 h, infected cells were selected for 72 h with puromycin (2 µg/ml).

### Cell proliferation assay

An equal number of cells were plated to 96-well plates. Cell proliferation was monitored by a 3-(4, 5-dimethylthiazol-2-yl)-2, 5-diphenyltetrazolium bromide (MTT) Cell Proliferation/Viability Assay kit (BioVision) according to the guidelines.

### Cellular colony formation assay

The glioma cells were suspended in 1.5 ml complete medium, which was pre-supplemented with low melting point agarose (Invitrogen) at the concentration of 0.45%. Then the cells were plated in 35 mm tissue culture plates containing 1.5 ml complete medium (ThermoFisher) and 0.6% agarose (Sigma) on the bottom layer. The cells were cultured at 37 °C with 5% CO_2_ for 2 weeks and the culture medium was replaced every 3 days. Finally, the formed cell colonies were stained with the crystal violet (Beyotime Biotechnology, 0.005%) and analyzed using a microscope. The colony number in each well was calculated and relative colony formation capacity was shown.

### Xenograft experiment

For subcutaneous xenograft models, the experiments were performed based on a previous publication [19]. Briefly, an equal number (10^7^ cells per mouse) of U87MG cells with/without JMJD2A knockdown were implanted subcutaneously into the left flanks of 8-week-old nude mice. The growth of the tumor was monitored by measurement of the lengths and widths of tumors, and the tumor volume was calculated. At the end of the experiment, the tumors were harvested and tumor weight was analyzed. The animal study has been approved by the Animal Use and Care Committee of the General Hospital of Western Theater Command.

### Protein synthesis assay

Glioma cells were infected with lentivirus expressing sh-JMJD2A or control shRNA for 24 h. Then the [^3^H]-leucine was added to the culture medium to reveal the protein synthesis. Incorporation of [^3^H]-leucine into total cellular protein was determined 24 h later and results were normalized to the DNA content of the cells.

### Chromatin immunoprecipitation (ChIP)

ChIP assay was performed using the kit Chromatin Immunoprecipitation (ChIP) Assay Kit (Abcam). The enrichment of JMJD2A, H3K9me3 and H3K36me3 at PDK1 gene promoter was analyzed with qRT-PCR, the relative enrichment was normalized with IgG control and Input. The enrichment at chromatin was analyzed by qRT-PCR. For the control distal region (Primer #1), the primers used for qRT-PCR were: forward (5′-ACCCAACTGTCTCTTGGCCT-3′), reverse (5′-GGGAAAGGTAGGCTGTGTCAG-3′). For promoter region #1 (Primer #2), the primers used for qRT-PCR were: forward (5′-CCAACTTAGGTCCCGCAGAA-3′), reverse (5′-ATCTTTGCTGAGTGCCCGAG-3′). For promoter region #2 (near TSS site, primer #3, the primers used for qRT-PCR were: forward (5′-CGCGTTTGGATTCCGTG-3′), reverse (5′-CCAGTTATAATCTGCCTTCCCTATTATC-3′). For transcriptional region (Primer #4), the primers used for qRT-PCR were: forward (5′-TGCTGTATGGCCTGCAAGAT-3′), reverse (5′-ACATTCTGGCTGGTGACAGG-3′).

### Statistical analysis

All values are expressed as the mean ± SEM of at least three independent experiments. Statistical differences among groups were determined using either Student’s *t* test or one-way analysis of variance (ANOVA). Kaplan–Meier curve log-rank was performed to analyze the survival correlation with JMJD2A expression. repeated one-way ANOVA was performed to analyze the growth curve of tumors in vivo. *P* values of less than 0.05 were considered statistically significant. The analyses were performed using GraphPad Prism 6 software.

## Results

### JMJD2A is overexpressed in human glioma

**Fig. 1:**
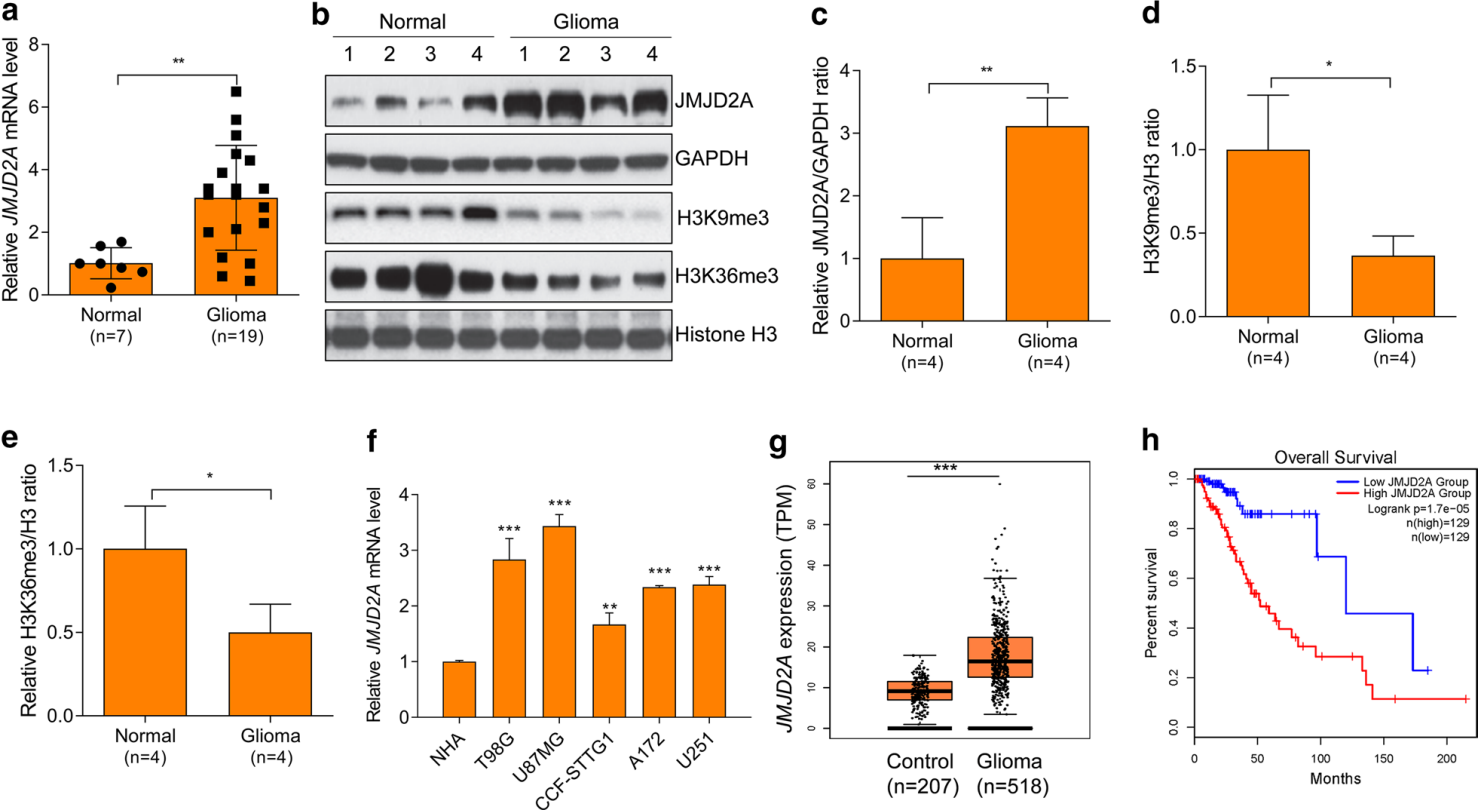
JMJD2A is up-regulated in human glioma. **a***JMJD2A* mRNA level in normal control brain tissues (n = 7) and human glioma tissues (n = 19). **p < 0.01. **b** JMJD2A, H3K9me3, H3K36me3 protein level in normal control brain tissues (n = 4) and human glioma tissues (n = 4). GAPDH and histone H3 were loading controls. **c** Quantitative results of the JMJD2A protein level in (**b**). *p < 0.05. n = 4 in each group. **d** Quantitative results of H3K9me3 protein level in (**b**). *p < 0.05. n = 4 in each group. **e** Quantitative results of H3K36me3 protein level in (**b**). *p < 0.05. n = 4 in each group. **f** JMJD2A mRNA level in human normal astrocyte cells (NHA) and glioma cell lines (T98G, U251, U87MG, A172, and CCF-STTG1). *p < 0.05, ** *p *< 0.01 *vs.* NHA. **g** Expression of JMJD2A in glioma and control normal tissues analyzed with GEPIA. ***p < 0.001. **h** Survival analysis (Kaplan–Meier curve) of high JMJD2A expression (top 25%) and low JMJD2A expression (bottom 25%) glioma patients. Log-rank *p *= 1.7E–5

In the present study, we aimed to investigate the potential role of JMJD2A in human glioma. Firstly, we explored the expression pattern of JMJD2A mRNA and protein in 7 normal brain tissues and 19 glioma tissues. We found that JMJD2A mRNA was much higher in tissues from glioma than that from normal brain tissues (Fig. 1a). Consistently, western blot revealed that the protein level of JMJD2A was up-regulated in glioma tissues (Fig. 1b, c). JMJD2A is a JmjC histone demethylase (HDM) that catalyzes the demethylation of H3K9me2/3 and H3K36me2/3 [20]. Therefore, we also studied the protein level of H3K9me3 and H3K36me3 in normal brain and glioma tissues. In consistent with the up-regulation of JMJD2A, the levels of H3K9me3 and HEK36me3 were significantly down-regulated in glioma compared with normal brain tissues (Fig. 1b, d, e). Furthermore, we also investigated the expression of JMJD2A in one normal human astrocyte cell line (NHA) and five human glioma cell lines (T98G, U87MG, U251, A172, and CCF-STTG1). The results showed that the mRNA of JMJD2A was much higher in glioma cell lines compared with that in astrocyte cells (Fig. 1f). In addition, we also analyzed the expression and correlation with the survival of JMJD2A in glioma using data from the TCGA database with the web tool GEPIA2. 207 controls and 518 gliomas were analyzed and we observed that JMDJ2A expression was significantly higher in glioma than the control and that high expression of JMJD2A predicted poor overall survival (Fig. 1g, h). Taken together, the expression of JMJD2A is overexpressed in human glioma.

### JMJD2A promotes glioma cell growth and transformation

**Fig. 2:**
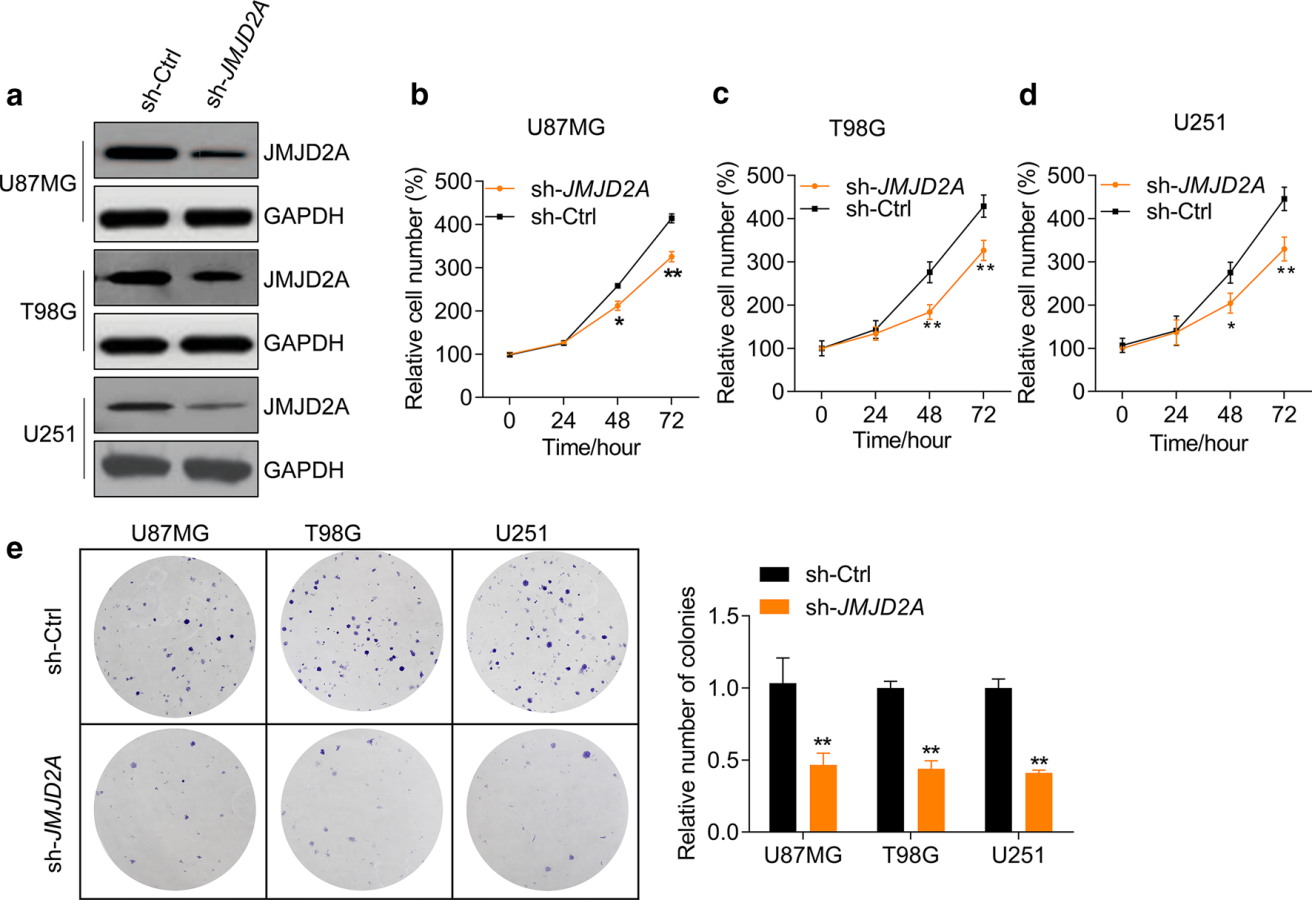
JMJD2A knockdown reduces glioma cell growth and colony formation. **a***JMJD2A* knockdown with lentivirus-mediated shRNA in U87MG, T98G, and U251 glioma cells. glioma cells were infected with lentivirus expressing sh-Ctrl or sh-JMJD2A for 48 h. (**b**–**d**) *JMJD2A* knockdown represses the proliferation of U87MG (**b**), T98G (**c**) and U251 (**d**) glioma cells. Glioma cells were infected with lentivirus expressing sh-ctrl or sh-*JMJD2A* and cell number was determined at indicated time points. *p < 0.05, **p < 0.01 *vs.* sh-ctrl. **e***JMJD2A* knockdown inhibits colony formation of glioma cells. glioma cells with/without *JMJD2A* knockdown were subjected to colony formation assay. The cells were cultured for 2 weeks and the colony number was determined. **p < 0.01 *vs.* sh-ctrl

**Fig. 3:**
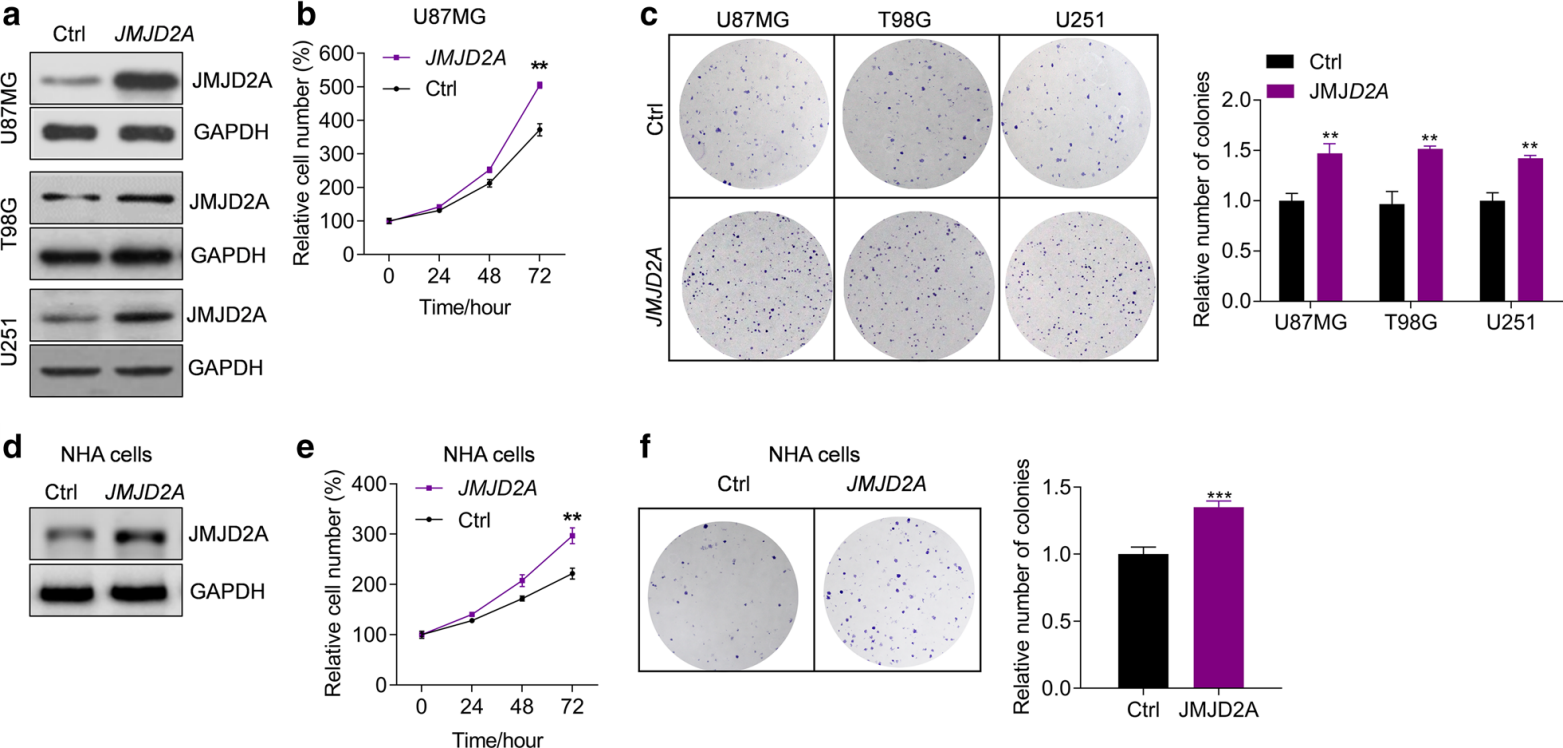
JMJD2A overexpression promotes cell growth and colony formation. **a** Lentivirus-mediated *JMJD2A* overexpression in glioma cells. Glioma cells were infected with lentivirus expressing ctrl or JMJD2A for 48 h. **b***JMJD2A* overexpression promotes the proliferation of U87MG glioma cells. U87MG cells were infected with lentivirus expressing ctrl or *JMJD2A* and cell number was determined at indicated time points. **p < 0.01 *vs.* ctrl. **c***JMJD2A* overexpression facilitates colony formation of glioma cells. Glioma cells with/without *JMJD2A* overexpression were subjected to colony formation assay. The cells were cultured for 2 weeks and the colony number was determined. **p < 0.01 *vs.* ctrl. **d** Lentivirus-mediated *JMJD2A* overexpression in NHA cells. NHA cells were infected with lentivirus expressing ctrl or JMJD2A for 48 h. **e***JMJD2A* overexpression promotes the proliferation of NHA cells. NHA cells were infected with lentivirus expressing ctrl or *JMJD2A* and cell number was determined at indicated time points. **p < 0.01 *vs.* ctrl. **f***JMJD2A* overexpression facilitates colony formation of NHA cells. NHA cells with/without *JMJD2A* overexpression were subjected to colony formation assay. The cells were cultured for 2 weeks and the colony number was determined. ***p < 0.001 *vs.* ctrl

The high expression pattern of JMJD2A prompted us to study its potential role in glioma cell growth. To this end, we knocked down JMJD2A with lentivirus-mediated shRNA in glioma cell U87MG, T98G, and U251 cells, as validated by western blot (Fig. 2a). Then we investigated the effects of JMJD2A on glioma cell proliferation and colony formation. The results demonstrated that JMJD2A knockdown repressed the proliferation rate of U87MG, T98G and U251 cells (Fig. 2b–d). Transformation is a hallmark of cancer cells; therefore, we analyzed the effects of JMJD2A on the transformation of glioma cells with colony formation assay. Similarly, the colony formation activity of glioma cells was also inhibited by JMJD2A shRNA (Fig. 2e). These results implicate that JMJD2A low-expression reduces the proliferation and colony formation in glioma cells. We further overexpressed JMJD2A with lentivirus in glioma U87MG cells and found that JMJD2A overexpression promoted U87MG cell proliferation and colony formation (Fig. 3a, b). Consistently, the colony formation capacity of T98G and U251 cells were enhanced when JMJD2A was overexpressed (Fig. 3c**)**. Consistently, JMJD2A overexpression in NHA cells promoted cell proliferation and colony formation (Fig. 3d–f). Altogether, JMJD2A promotes glioma cell proliferation and colony formation.

### JMJD2A promotes glioma in vivo

**Fig. 4:**
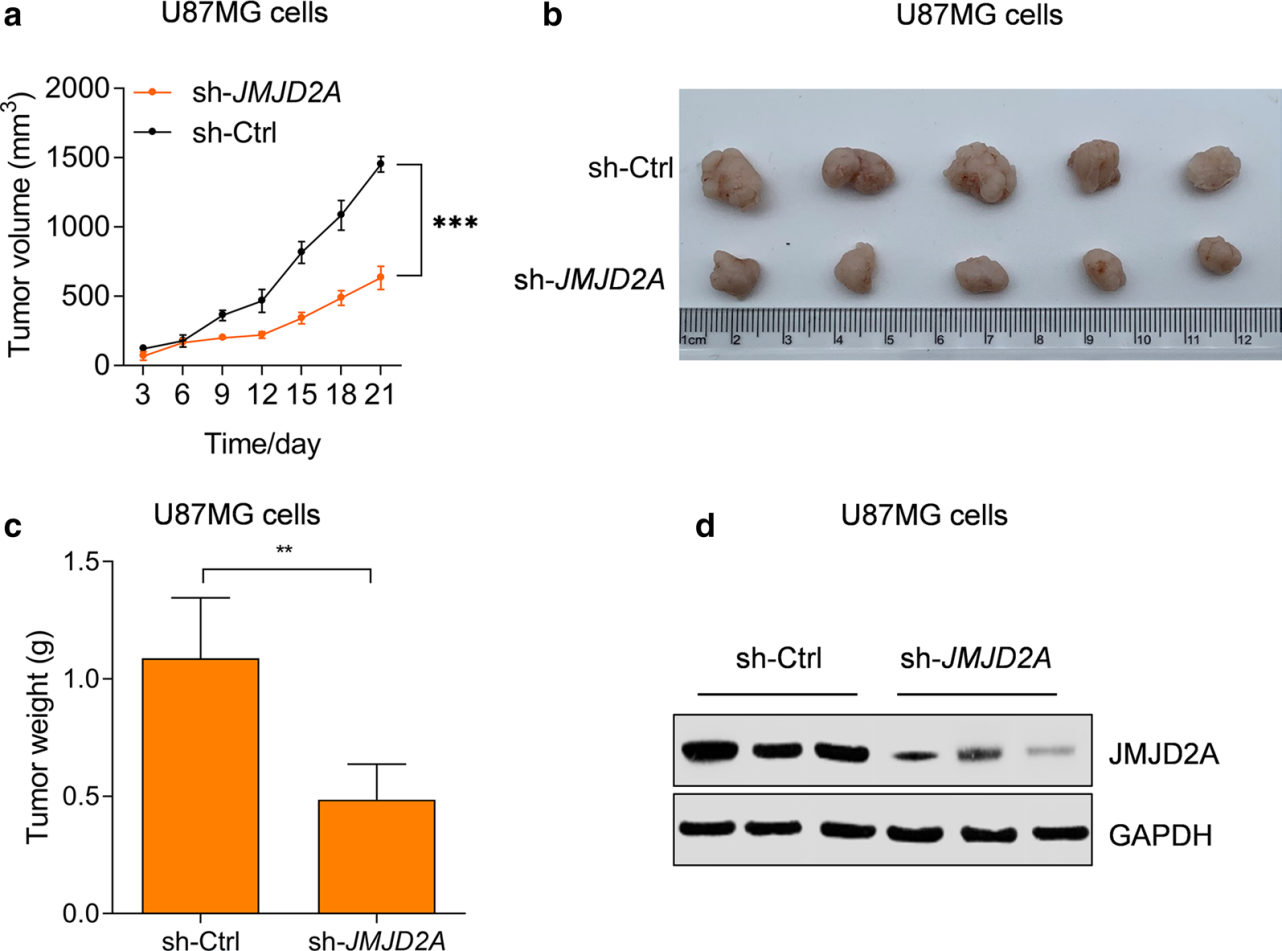
JMJD2A facilitates the growth of glioma in vivo. **a**–**c***JMJD2A* knockdown represses U87MG growth in vivo. Equal numbers of T98G cells with/without JMJD2A knockdown were subjected to xenograft experiments. **a** Growth curve of glioma U87MG cells. **b** Representative pictures of tumors. **c** Tumor weight. **p < 0.01, ***p < 0.001. n = 5 in each group. **d** Western blot showing the expression of JMJD2A in tumor tissues

To further explore whether JMJD2A also promoted glioma cell growth in vivo, we performed a xenograft experiment. U87MG cells with/without sh*JMJD2A* transduction were used for xenograft experiments. The growth curve revealed that the *JMJD2A* knockdown repressed the growth rate of U87MG cells in vivo (Fig. 4a). In addition, *JMJD2A* knockdown reduced the final size and weight of glioma tumors in vivo (Fig. 4b, c). In addition, the western blot also confirmed that the expression of JMJD2A was reduced in tumor tissues from the JMJD2A knockdown group (Fig. 4d). Taken together, these findings demonstrated that JMJD2A regulated glioma growth in vivo.

### JMJD2A regulates Akt-mTOR and protein synthesis

**Fig. 5:**
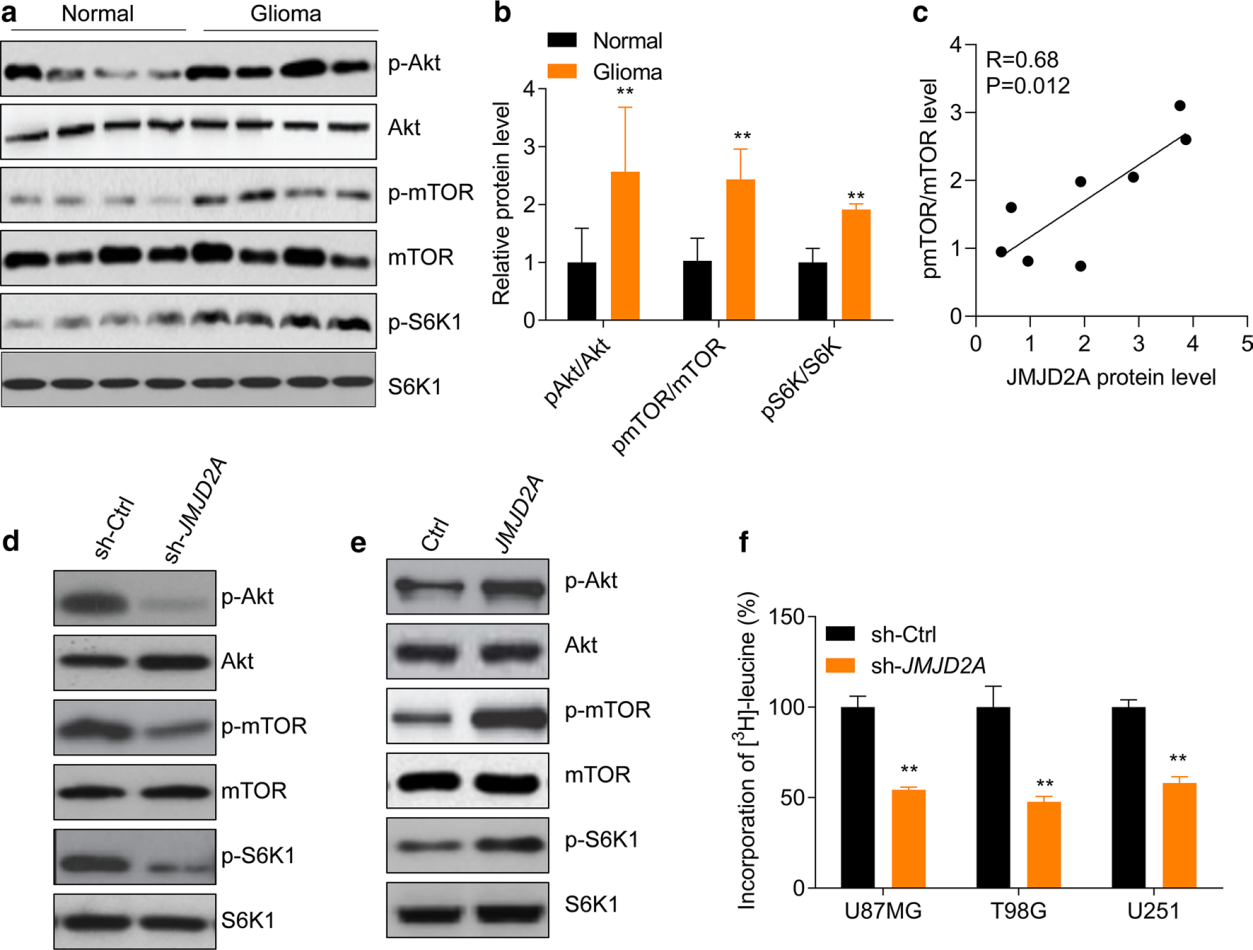
JMJD2A facilitates the activation of the Akt-mTOR pathway in glioma. **a** Akt-mTOR pathway is activated in human glioma tissues (n = 4) compared with the control (n = 4). **b** Quantitative results of the protein levels in (**a**). **p < 0.01 *vs.* Normal. n = 4 in each group. **c** Correlation analysis of JMJD2A protein level and mTOR activation in glioma tissues and control brain tissues (n = 8). **d***JMJD2A* knockdown represses the activation of the Akt-mTOR pathway in U87MG glioma cells. U87MG cells were infected with lentivirus expressing sh-Ctrl or sh-JMJD2A for 48 h. **e***JMJD2A* overexpression promotes the activation of the Akt-mTOR pathway in U87MG glioma cells. U87MG cells were infected with lentivirus expressing ctrl or *JMJD2A* for 48 h. **f***JMJD2A* knockdown represses protein synthesis in glioma cells. U87MG, T98G, and U251 cells were infected with lentivirus expressing sh-ctrl or sh-JMJD2A for 24 h and then protein synthesis assay was performed with the incorporation of [^3^H]-leucine for 24 h. **p < 0.01 *vs.* sh-ctrl

Serving as a central signaling hub integrating multiple intra- and extra-cellular cues, the serine/threonine kinase mammalian target of rapamycin (mTOR) is an attractive anticancer target [21]. mTOR is involved in the formation of at least two multi-protein complexes, mTORC1, and mTORC2, that direct cell metabolism, growth, proliferation, survival, and angiogenesis [21]. The rapamycin-sensitive mTORC1 essentially mediates phosphoinositide-3-kinase (PI3K)/protein kinase B (AKT/PKB) signals. Furthermore, through its direct phosphorylation of the ribosomal protein S6 kinase 1 (S6K1) and the eukaryotic translation initiation factor 4E binding protein 1, the mTORC1 promotes anabolic processes, including biosynthesis of proteins, lipids, and organelles, and limits catabolic processes such as autophagy [22]. The role of mTOR in children and adult glioma was widely investigated [23, 24]. We found that the Akt-mTOR pathway is hyperactivated in glioma tissues (Fig. 5a, b), where the expression of JMJD2A was also high (Fig. 1a–c), indicating that JMJD2A is related to mTOR activation. Indeed, we observed that JMJD2A expression was positively correlated with mTOR activation by performing linear correlation analysis (Fig. 5c). We hypothesized that JMJD2A may regulate the activation of mTOR in glioma cells. Therefore, we knocked down JMJD2A in human U87MG cells and found that JMJD2A knockdown reduced the levels of p-Akt, p-mTOR, and p-S6K (Fig. 5d). In contrast, JMJD2A overexpression promoted the activation of the Akt-mTOR-S6K pathway in U87MG cells (Fig. 5e). We also found that JMJD2A knockdown reduced the protein synthesis in U87MG, T98G and U251 glioma cells (Fig. 5f). Taken together, JMJD2A regulates the activation of the Akt-mTOR pathway and protein synthesis in human glioma cells.

### Inhibition of mTOR blocks the effects of JMJD2A

**Fig. 6:**
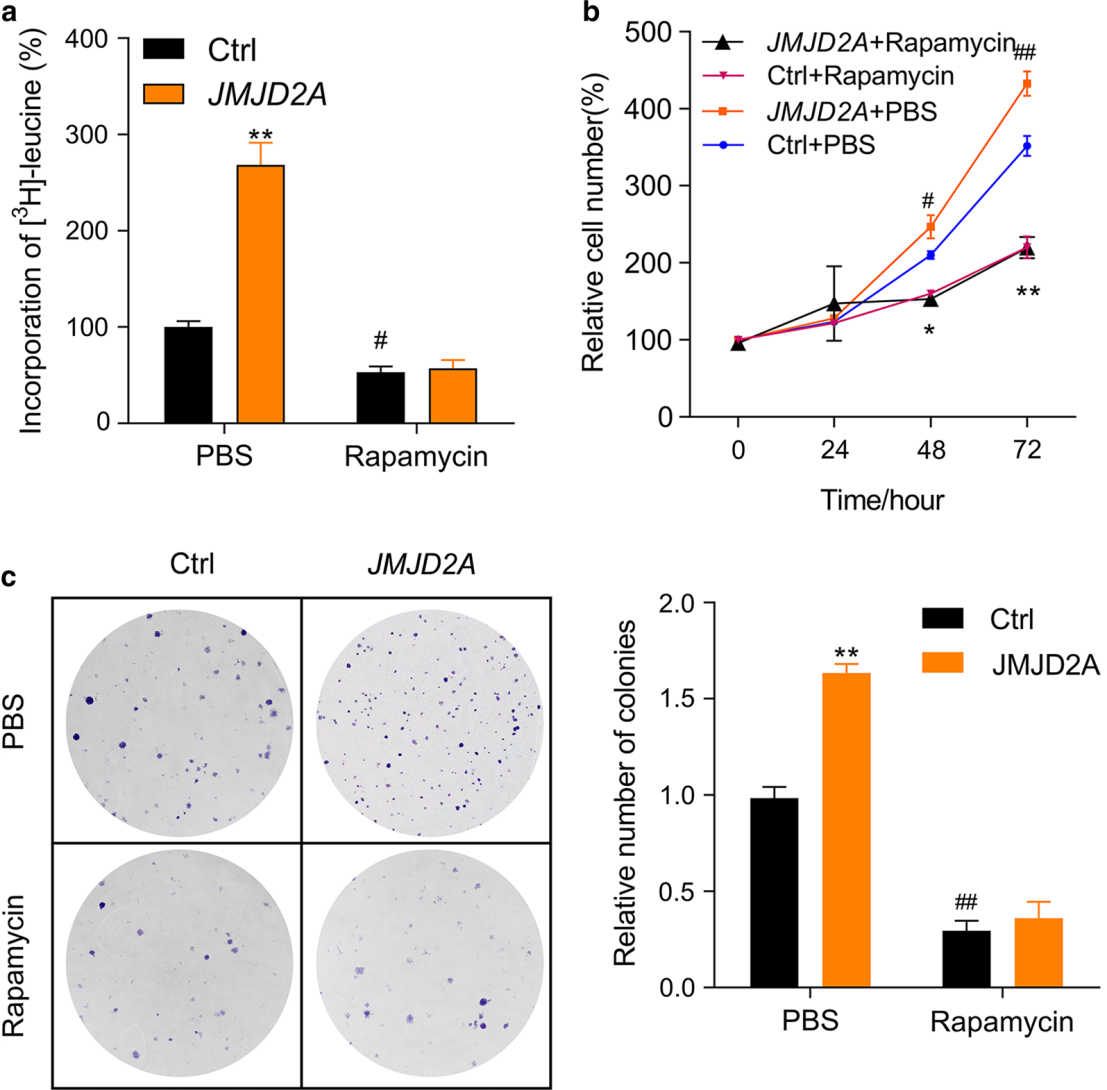
Rapamycin blocks the effects of JMJD2A on glioma cell growth and colony formation. **a** Inhibition of mTOR with rapamycin blocks the effects of JMJD2A on protein synthesis in U87MG glioma cells. U87MG cells were pretreated with rapamycin (1 μM) for 12 h followed by lentivirus infection and protein synthesis assay. **p < 0.01 *vs.* ctrl + PBS; #p < 0.05 *vs.* ctrl + PBS. **b** Inhibition of mTOR with rapamycin blocks the effects of JMJD2A on the proliferation of U87MG cells. U87MG cells were pretreated with rapamycin (1 μM) for 12 h followed by lentivirus infection and proliferation assay. *p < 0.05, **p < 0.01 *vs.* Ctrl + PBS; #*p *< 0.05, ##*p *< 0.01 *vs*. ctrl + PBS. **c** Inhibition of mTOR with rapamycin blocks the effects of JMJD2A on colony formation of U87MG cells. U87MG cells with/without JMJD2A overexpression were subjected to colony formation assay in the presence/absence of rapamycin (1 μM). **p < 0.01 *vs.* ctrl + PBS; ##p < 0.01 *vs.* ctrl + PBS

As we have demonstrated that JMJD2A regulated the activation of Akt-mTOR signaling, we wanted to know whether mTOR is essential for the function of JMJD2A. To this end, we inhibited mTOR signaling with its inhibitor rapamycin in U87MG cells. The results showed that rapamycin-mediated mTOR inhibition reduced the protein synthesis in glioma cell U87MG and blocked the effect of JMJD2A overexpression on protein synthesis (Fig. 6a). Furthermore, rapamycin markedly reduced proliferation rate and colony formation of U87MG cells (Fig. 6b, c). However, when mTOR was inhibited with rapamycin, JMJD2A overexpression could not affect the proliferation and colony formation of glioma cells (Fig. 6b, c). These findings indicate that mTOR signaling contributes essentially to the role of JMJD2A in human glioma cells.

### JMJD2A activates Akt-mTOR by promoting PDK1 expression

**Fig. 7:**
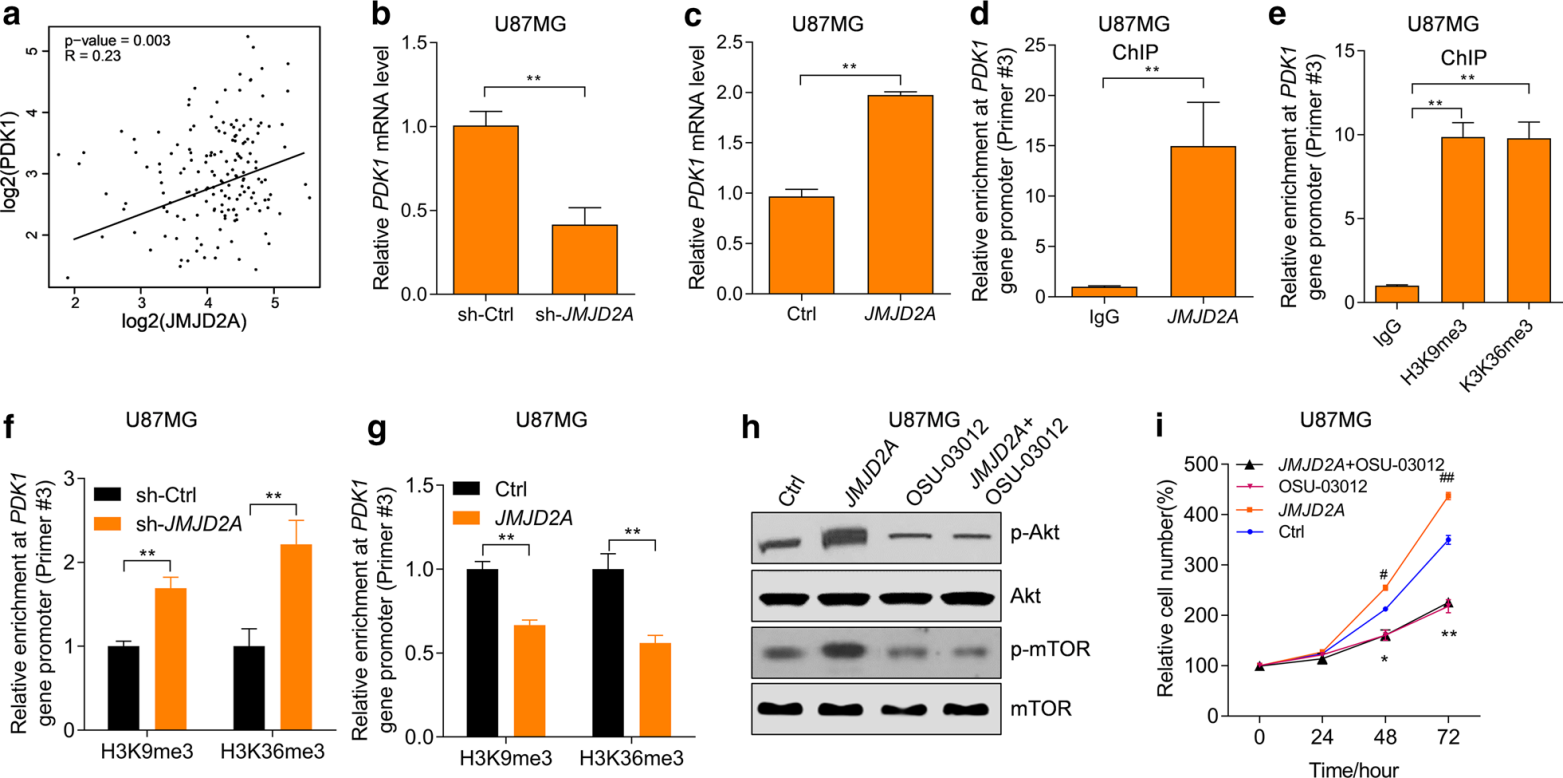
JMJD2A activates PDK1 to regulate Akt-mTOR signaling. **a** JMJD2A expression is correlated with PDK1 expression in glioma tissues based on the TGCA database. **b** JMJD2A knockdown reduces PDK1 expression in U87MG cells. U87MG cells were infected with lentivirus expressing sh-ctrl or sh-JMJD2A for 48 h. **p < 0.01. **c** JMJD2A overexpression promotes PDK1 expression in U87MG cells. U87MG cells were infected with lentivirus expressing ctrl or *JMJD2A* for 48 h. **p < 0.01. **d** Chromatin immunoprecipitation assay demonstrates the enrichment of JMJD2A on the PDK1 promoter in U87MG cells. **p < 0.01. **e** Chromatin immunoprecipitation assay demonstrates the enrichment of H3K9me3 and H3K36me3 n the PDK1 promoter in U87MG cells. **p < 0.01. **f** JMJD2A knockdown increases the enrichment of H3K9me3 and H3K36me3 at PDK1 promoter in U87MG cells. U87MG cells were infected with lentivirus for 48 h. **p < 0.01. **g** JMJD2A overexpression reduces the enrichment of H3K9me3 and H3K36me3 at PDK1 promoter in U87MG cells. U87MG cells were infected with lentivirus for 48 h. **p < 0.01. **h** Inhibition of PDK1 with OSU-03012 blocks JMJD2A-mediated activation of Akt-mTOR signaling. The U87MG cells with/without JMJD2A overexpression were treated with OSU-03012 (1 μM) for 24 h. **i** Inhibition of PDK1 with OSU-03012 blocks JMJD2A-mediated effects on the proliferation of U87MG cells. U98MG with/without JMJD2A overexpression were treated with OSU-03012 (1 μM)

Since we observed that JMJD2A activates AKT-mTOR signaling, we hypothesized that JMJD2A regulates the upstream regulator of AKT. Phosphoinositide-dependent kinase-1 (PDK1) is a regulator of AKT-mTOR signaling. Activation of the upregulation of PDK1 promotes the activation of AKT-mTOR signaling in cancer cells [25]. JMJD2A (KDM4A) was reported to coactivate with E2F1 to regulate the PDK1-dependent metabolic switch [26]. Therefore, we hypothesized that JMJD2A activates AKT-mTOR signaling by promoting the expression of PDK1. Indeed, we observed that JMJD2A expression was positively correlated with *PDK1* expression in glioma tissues (Fig. 7a). In addition, loss-of-function and gain-of-function experiments showed that JMJD2A promoted *PDK1* expression in glioma cells (Fig. 7b, c). We next investigated whether JMJD2A bound *PDK1* promoter and regulated histone methylation. To this end, chromatin immunoprecipitation (ChIP) assay was performed and enrichment of JMJD2A, H3K9me3 and H3K36me3 at *PDK1* promoter was observed in U87MG cells (Fig. 7d, e, Additional file 1: Figure S1a, b). JMJD2A was enriched at the *PDK1* promoter but not distal or transcriptional regions (Additional file 1: Figure S1a, b). Of note, JMJD2A knockdown increased whereas JMJD2A overexpression reduced the enrichment of H3K9me3 and H3K36me3 at *PDK1* promoter (Fig. 7f, g). Therefore, JMJD2A bound *PDK1* promoter and demethylate H3K9 and H3K36 to activate the expression of *PDK1*. To further explore whether PDK1 was critically involved in JMJD2A-mediated activation of AKT-mTOR, we inhibited PDK1 with its inhibitor OSU-03012. OSU-03012 treatment repressed AKT-mTOR activation and blocked the effects of JMJD2A on AKT-mTOR activation (Fig. 7h). Finally, we provided evidence that inhibition of PDK1 with OSU-03012 reduced glioma cell proliferation and blocked the effects of JMJD2A overexpression (Fig. 7i). Taken together, these findings demonstrated that JMJD2A epigenetically regulated the expression of PDK1 to activate Akt-mTOR signaling and promote glioma cell proliferation.

## Discussion

In this study, we showed that the histone demethylase JMJD2A was up-regulated in glioma. The high level of JMJD2A in glioma was associated with a low level of H3K9me3 and H3K36me3 as well as hyperactivation of the Akt-mTOR signaling pathway. High expression of JMJD2A predicted poor overall survival. In glioma cells, we used *loss*-*of*-*function* and *gain*-*of*-*function* experiments to demonstrate that JMJD2A promotes proliferation and colony formation of glioma U87MG, T98G, and U251 cells, and U87MG growth in vivo. JMJD2A activated Akt-mTOR signaling pathway and regulated protein synthesis in glioma cells via promoting PDK1 expression. Additionally, we provided evidence that the effects of JMJD2A on protein synthesis, cell proliferation, and colony formation were blocked by rapamycin, implicating that mTOR is essential for JMJD2A function in human glioma cells.

High-grade gliomas are typically treated with a combination of surgery, radiotherapy and/or chemotherapy [27]. Given the high mortality and poor prognosis of glioma patients, there is a significant need for the development of more efficient therapeutic strategies, in which epigenetic targets could be a good option for the mission. Histone methylation has been reported to be an important factor that affects the development of glioma. Recent studies have identified a Lys 27-to-methionine (K27M) mutation at one allele of H3F3A, one of the two genes encoding histone H3 variant H3.3, in 60% of high-grade glioma cases [28–30]. The histone H3.3K27M mutation in glioma reprograms H3K27 methylation and gene expression [4]. Reduced H3K27me3 and DNA hypomethylation are major drivers of gene expression in K27M mutant high-grade gliomas [3]. Here in this study, we found that H3K9m3 and H3K36me3 were down-regulated in gliomas, which may result from hyper-expression of the histone demethylase JMJD2A. JMJD2A was overexpressed in human gliomas and glioma cell lines, indicating that JMJD2A may serve as a biomarker of pediatric high-grade gliomas. Importantly, we observed that JMJD2A high expression was associated with poor overall survival in glioma patients. Further study is needed to explore whether the expression level of H3K9m3/H3K36me3 is associated with the prognosis of gliomas.

In three glioma cell lines, we utilized *loss*-*of*-*function* and *gain*-*of*-*function* experiments by knocking down or overexpressing JMJD2A with lentivirus and found that JMJD2A promoted glioma cell proliferation and colony formation and that JMDJ2A promoted glioma cell growth in vivo. These biological functions of JMJD2A relied on its effects on protein synthesis. We showed that JMJD2A promoted protein synthesis by activating the Akt-mTOR pathway in pediatric high-grade gliomas tissues and glioma cell lines. Inhibition of mTOR with rapamycin blocked the effects of JMJD2A on protein synthesis, cell proliferation, and colony formation, indicating that the mTOR pathway is important for the role of JMJD2A in glioma. The role of mTOR is fully understood in adult glioma [22], and a recent study demonstrated that activation of mTORC1/mTORC2 signaling in pediatric low-grade glioma [24]. Here we first show that mTOR was also activated in glioma and that JMJD2A was an upstream driver of the mTOR pathway. A current report also JMJD2A depletion reduced protein synthesis and enhanced the protein synthesis suppression observed with mTOR inhibitors, which paralleled an increased sensitivity to drugs [31], indicating that JMJD2A may serve as an adjuvant target for protein synthesis inhibition in glioma. Nevertheless, we further should investigate how the cytoplasmic mTOR senses the nucleus JMJD2A signal in glioma. Previous work showed that JMJD2A (KDM4A) repressed mTOR activation by maintaining the stability of mTOR repressor DEPTOR (DEP domain-containing mTOR-interacting protein) [32]. JMJD2A is a histone demethylase. Although previous work found that JMJD2A maintaining DEPTOR stability, the underlying mechanism may be indirect. The mechanism by which JMJD2A maintaining the stability of DEPTOR remains to elucidate. In addition, all the experiments of that previous work were performed in Hela cells. Whether JMJD2A maintains DEPTOR stability in other types of cells remains to study. Interestingly, our mechanism study provided direct evidence that JMJD2A promoted the expression of PDK1 to activate Akt-mTOR signaling. JMJD2A directly bound *PDK1* promoter, where this histone demethylase reduced the enrichment of H3K9me3 and H3K36me3 to activate the expression of PDK1. PDK1 is an activator of the AKT-mTOR signaling axis [25]. In addition, the role of JMJD2A in regulating metabolic switch also relied on the activation of PDK1 [26]. Taken together, these findings may also implicate that JMJD2A is involved in metabolism and that the effects of JMJD2A on the mTOR signaling is complex and multidimensional.

H3K36me3 normally shows on the gene body (transcriptional region) to activate gene expression [33]. Interestingly, the enrichment of H3K36me3 at the promoter of mammalian and plant genes were also observed [34, 35]. For instance, H3K36me3 is enriched at the *Protamine 2 (PRM2)* promoter to promote *RRM2* expression in U2OS cells [34]. Herein this study, JMJD2A and H3K9me3 were enriched at the promoter of the *PDK1* gene, while H3K36me3 was enriched around the transcriptional start site (TSS) and transcriptional region of *PDK1* gene ( Additional file 1: Figure S1a). Although JMJD2A may regulate H3K36me3 around the TSS site of the *PDK1* gene, JMJD2A was not enriched at the transcriptional region of the *PDK1* gene to regulate H3K36me3. Instead, our data revealed that JMJD2A bound at the promoter region of the *PDK1* gene to modulate the enrichment of H3K9me3 to promote the expression of the *PDK1* gene in glioma cells. As thus, H3K9me3 and H3K39me3 may share some regions at *PDK1* gene locus and JMJD2A targets H3K9me3 to promote the expression of *PDK1* in glioma cells. Further ChIP-seq may help to understand the regulation of PDK1 expression by H3K9me3 and H3K36me3.

## Conclusions

Here in this study, we demonstrate that the histone demethylase JMJD2A is overexpressed in human glioma. JMJD2A high expression is correlated with low levels of H3K9me3 and H3K36me3 and poor overall survival of glioma patients. JMJD2A promotes glioma cell growth by activating the Akt-mTOR pathway. These findings indicate that JMJD2A may be a potential target for therapy of glioma.

## Supplementary information

**Additional file 1: Figure S1.** Enrichment of JMJD2A, H3K9me3, and H3K36me3 at the gene locus of *PDK1.*

## Data Availability

All the data in the manuscript are available upon reasonable request.

## Abbreviations

JMJD2A: JmjC Domain-containing 2A
mTOR: Mammalian target of rapamycin
PI3K: Phosphoinositide-3-kinase
S6K1: S6 Kinase 1 (S6K1)
PDK1: Pyruvate dehydrogenase kinase 1
