# Supplementary material for: The histone demethylase JMJD2A promotes glioma cell growth via targeting Akt-mTOR signaling
Source: Cancer Cell Int. 2020 Mar 30;20:101. doi: 10.1186/s12935-020-01177-z (PMC7106579; doi:10.1186/s12935-020-01177-z)
Supplement: Supplementary file 1 — Additional file 1: Figure S1. Enrichment of JMJD2A, H3K9me3, and H3K36me3 at the gene locus of PDK1. [file 12935_2020_1177_MOESM1_ESM.docx]

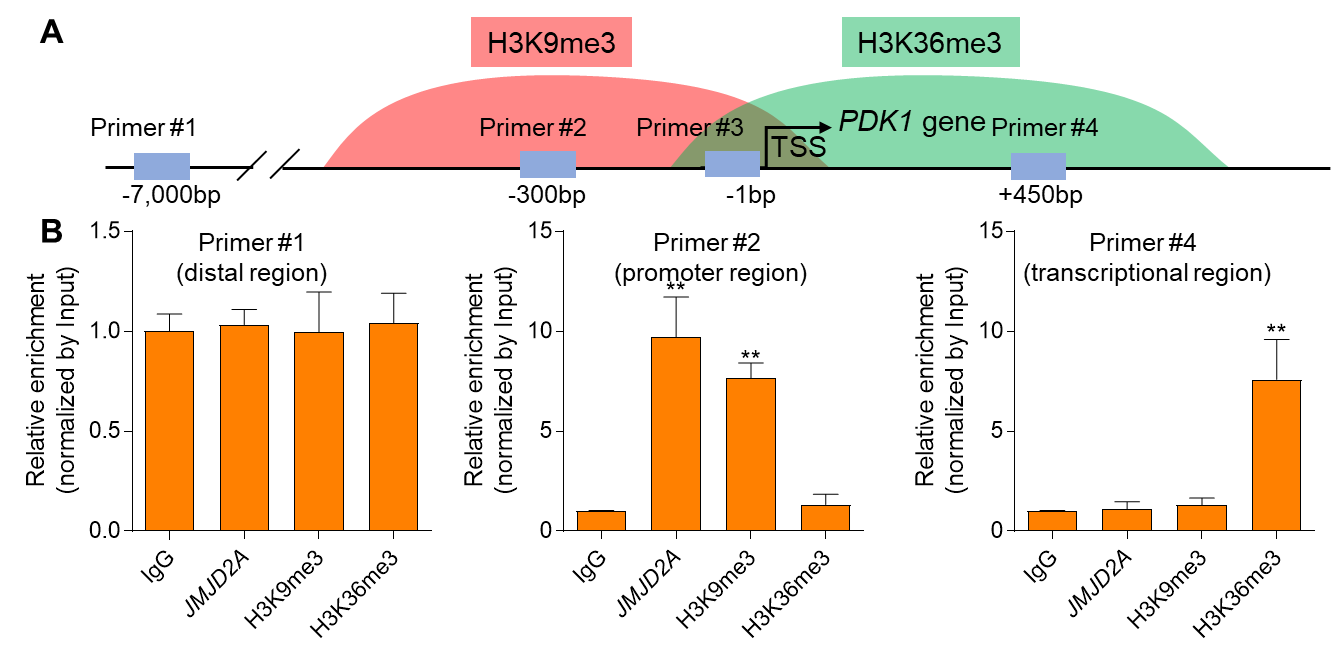


**Additional file 1: Figure S1 Enrichment of JMJD2A, H3K9me3, and H3K36me3 at the gene locus of *PDK1*.**

1. Design of primers for Qrt-PCR analysis of immunoprecipitated chromatins.
2. Enrichment of JMJD2A, H3K9me3, and H3K36me3 at the distal, promoter and transcriptional regions at *PDK1* gene locus. ** p<0.01 *vs.* IgG.
